# Dinitroaniline herbicides: a comprehensive review of toxicity and side effects on animal non-target organisms

**DOI:** 10.1007/s11356-022-23169-4

**Published:** 2022-09-29

**Authors:** Anita Giglio, Maria Luigia Vommaro

**Affiliations:** Department of Biology, Ecology and Earth Science, University of Calabria, via Bucci, 87036 Rende, Italy

**Keywords:** Apoptosis, Carcinogenesis, Ecotoxicology, Endocrine disruption, Histopathology, Microtubules, Pesticides, Survival rate

## Abstract

The widespread use of herbicides has increased concern about the hazards and risks to animals living in terrestrial and aquatic ecosystems. A comprehensive understanding of their effective action at different levels of biological organization is critical for establishing guidelines to protect ecosystems and human health. Dinitroanilines are broad-spectrum pre-emergence herbicides currently used for weed control in the conventional agriculture. They are considered extremely safe agrochemicals because they act specifically on tubulin proteins and inhibit shoot and root growth of plants. However, there is a lack of toxicity information regarding the potential risk of exposure to non-target organisms. The aim of the present review is to focus on side effects of the most commonly used active ingredients, e.g. pendimethalin, oryzalin, trifluralin and benfluralin, on animal non-target cells of invertebrates and vertebrates. Acute toxicity varies from slightly to high in terrestrial and aquatic species (i.e. nematodes, earthworms, snails, insects, crustaceans, fish and mammals) depending on the species-specific ability of tested organisms to adsorb and discharge toxicants. Cytotoxicity, genotoxicity and activation of oxidative stress pathways as well as alterations of physiological, metabolic, morphological, developmental and behavioural traits, reviewed here, indicate that exposure to sublethal concentrations of active ingredients poses a clear hazard to animals and humans. Further research is required to evaluate the molecular mechanisms of action of these herbicides in the animal cell and on biological functions at multiple levels, from organisms to communities, including the effects of commercial formulations.

## Introduction

Herbicides are agrochemicals widely used for weed control to increase crop yield and quality. Their sale value accounted for about 48% of the total global pesticide market share in 2019 (De et al. 2014; Sharma et al. 2018). However, direct or indirect adverse effects of their intensive use on non-target species are becoming evident in both terrestrial and aquatic ecosystems (Sharma et al. 2018; Thanomsit et al. 2020). Sublethal doses of herbicides enter habitats surrounding croplands through drift, runoff and/or volatilisation and directly affect the vegetative and reproductive stages of native plants, resulting in structural changes of communities and a reduction in species richness and abundance (Boutin et al. 2014; Ferreira et al. 2017). On the other hand, their increased use has also led to reports of resistant weeds (Chen et al. 2021), due to mechanisms of non-target-site resistance to herbicides with different mode of action (Jugulam and Shyam 2019). As a result, the ecological dynamics of crop-associated species are affected at all trophic levels (primary and secondary consumers, decomposers) (Prosser et al. 2016; Kraus and Stout 2019), involving species that provide ecosystem services (Pleasants and Oberhauser 2013) such as pollination (Russo et al. 2020) and pest control species, i.e. phytophagous (Gutiérrez et al. 2020), weed feeders and predators (Norris and Kogan 2000; Lami et al. 2020). In addition, physiological and behavioural effects of exposure at the organism level have been noted in species that play important ecological roles in agroecosystems (Freemark and Boutin 1995; Gunstone et al. 2021), such as nematodes (Sánchez-Moreno et al. 2015), earthworms (Stellin et al. 2018), collembolans and isopods (Niemeyer et al. 2018), spiders (Michalková and Pekár 2009; Korenko et al. 2016) and insects (Prosser et al. 2016; Capinera 2018; Sharma et al. 2018). Furthermore, components of commercial formulations, including the active ingredient and surfactants, may enter the aquatic environment, through direct runoff and leaching, and impact species of aquatic communities (Capinera 2018; Klementová et al. 2019; Thanomsit et al. 2020; y García et al. 2022).

This review provides an overview of current evidence concerning the side effects of dinitroaniline herbicides on non-target animals, cross-referencing available results from the literature and considering large-scale consequences of exposure. Currently, the evaluated annual consumption of dinitroaniline in agricultural areas of the USA accounted for about 4500 tonnes in 2018 (US Geological Survey 2017), while sales in Europe were approximatively 400 tonnes in 2020 (representing the 4% of total pesticide sales) (Eurostat 2020). The extensive use of these herbicides has raised serious environmental concerns. Indeed, a recent study indicated that a significant amount of dinitroaniline residues (from 48 up to 6906 μg·kg^−1^), which exceed permitted levels, have been found in the soil of agricultural regions around the word, though they had been banned and had not been applied to land for years (Sabzevari and Hofman 2022). The European Food Safety Authority (EFSA) reports a low risk from exposure to some active ingredients of this herbicide class to the non-target soil mesofauna, macrofauna and microorganisms (EFSA 2016; EFSA et al. 2019). However, the persistence of dinitroaniline residues such as pendimethalin and benfluralin in the environment has a significant impact on the richness of bacteria (Strandberg and Scott-Fordsmand 2004) and fungi (Roca et al. 2009) of soil communities reducing their growth (Singh et al. 2002; Kocárek et al. 2016) by up to 61% from 25 days after treatment (Nayak et al. 1994), thereby affecting soil biodiversity and fertility. As a result, a better understanding of their potential ecotoxicological effects and mechanisms of action on the animal cellular compartment is required to assess the exposure risk at all levels of biological organisation and on human health.

The main objective of this study is to outline and discuss the state of the art in the accumulation rate and acute and chronic toxicity of dinitroanilines by comparing different active ingredients and organisms, in order to support future tactics to mitigate adverse effects on non-target organisms without compromising plant growth, pest control and food production.

## Mode of action

Dinitroanilines are aromatic compounds (C_6H5N3O4_), with low water solubility (lipophilic), that are mainly used as pre-emergence herbicides to control annual grasses and some broadleaf weeds. This class includes benfluralin, butralin, chlornidine, dinitramine, dipropalin, ethalfluralin, fluchloralin, isopropalin, methalpropalin, nitralin, nitrofor, oryzalin, pendimethalin, prodiamine, profluralin and trifluralin (Nyporko et al. 2009). Benfluralin and pendimethalin are actually the only active ingredients (a.i.) approved by EFSA for marketing in EU countries (European Commission Website 2021). Some active ingredients such as butralin, dinitramine, ethalfluralin, isopropalin, nitralin, nitrofor, oryzalin and trifluralin are currently not authorised because of a lack of data on their fate and behaviour in the field, and information on the risk to terrestrial and aquatic non-target organisms. Despite this, butralin, ethalfluralin, oryzalin and trifluralin are permitted by Environmental Protection Agency of the USA (EPA 2017). According to UE regulations, there are commercial formulations containing these herbicides as active ingredients, either alone or in combination with two other herbicides. The field rate admitted is from 2 to 4 L ha^−1^ of pendimethalin-based commercial formulations (38% of a.i.) and from 2 to 8 L ha^−1^ of benfluralin-based commercial formulations (19% of a. i.).

They are well-known compounds to bind tubulin dimers of plants (Vaughn and Lehnen 1991; Sheval et al. 2008; Rose et al. 2016), protozoa (Traub-Cseko et al. 2001; Fennell et al. 2006) and fungi (Qu et al. 2018), most likely interacting with alpha-tubulin and interfering with microtubule polymerisation (Morejohn et al. 1987; Anthony and Hussey 1999; Morrissette and Sept 2009; Nyporko et al. 2009). In *Chlamydomonas*, a beta-tubulin mutation (Lys-350-Glu/Met) conferring resistance to dinitroanilines indicates that beta-tubulins may also be a target of these herbicides (Lee and Huang 1990).

Microtubules (MTs) are composed of heterodimeric subunits consisting of an alpha- and beta-tubulin (Howard and Hyman 2003). They are an integral part of the cytoskeleton and participate in several movement-related processes, including chromosome migration in mitosis and meiosis and cytoplasmic transport of vesicles (Gunning and Hardham 1982; Janke and Magiera 2020).

In plants, the first mechanism of action reported for dinitroanilines is the disruption of the mitotic sequence forming a tubulin-dinitroaniline complex, while there are no effects on the G1, S or G2 phases of the cell cycle (Hess and Bayer 1974; Strachan and Hess 1983; Duke 1990). They cause the disappearance of the microtubule mitotic spindle in treated meristem cells, preventing normal chromosome segregation and the cell division inhibiting the root elongation (Bartels and Hilton 1973; Fernandes et al. 2013). A secondary mechanism by which dinitroanilines affect MTs was studied in vitro on mitochondria isolated from plant tissues (Hertel et al. 1980). Exposure to trifluralin and oryzalin causes inhibition of Ca^2+^ transport mechanisms at the plasmatic membrane, suggesting that the anti-MT properties are related to deregulation of cytoplasmatic Ca^2+^ levels (Fernandes et al. 2013).

## Persistence and bioaccumulation

Loss of dinitroaniline from soil includes volatilisation, photodegradation in the surface zone, and microbial and chemical degradation in the soil incorporation zone (Weber 1990; Grover et al. 1997; Strandberg and Scott-Fordsmand 2004). Their widespread and repeated annual use for weed control in agroecosystems has raised questions about the degree of translocation and persistence in soil (Belden et al. 2003, 2005). Physical and chemical properties highlight the low water solubility and high log Kow and Koc values of dinitroanilines (Table 1), indicating the compound’s tendency to adsorb to soil organic matter and suggesting its potential ability to bioaccumulate in living organisms (Vighi et al. 2017). For this reason, herbicides belonging to this class are considered to be non-mobile in soil and their leachability is considered to be low (Lewis et al. 2016). However, persistence has been demonstrated to depend on temperature, moisture, soil type, and aerobic and anaerobic conditions over time (Stoller and Wax 1977; Savage 1978; Poku and Zimdahl 1980; Rathod et al. 2010; White et al. 2015). Indeed, the half-life of pendimethalin changes depending on the physical and chemical conditions in aquatic or terrestrial compartments (EFSA 2016). In laboratory tests, a half-life of 24–39 to 76–98 days has been found in agricultural relevant soils under aerobic conditions at 20 °C (Vighi et al. 2017), and a residual dose (10–15%) remains in the soil for up to 300–400 days after physical, chemical or microbiological transformations (Strandberg and Scott-Fordsmand 2004). Trifluralin disappears from clay loam soil over a period of 105 days, rapidly in the initial phase of treatment and more slowly in the next phase, but it is completely degraded in 347 days at 30 °C and persists up to 951 days under low temperature conditions (10 °C) (Chowdhury et al. 2021). Moreover, the variability of the dissipation time in the soil has been shown to depend on the duration and number of field applications per year, as well as on the amount (kg ha^−1^ of active ingredient or commercial formulation) (Grover et al. 1997).

**Table 1:** Chemical and physical properties of dinitroanilines^a^

Dinitroaniline^b^	Molecular weight (g mol^−1^)	Vapor pressure (mm Hg)	Henry’s law constant (atm cu m/mol)	Solubility in water (mg L^−1^)	Octanol–water partition coefficient (log K_OW_)	Organic carbon partition coefficient(K_OC_)	Field dissipation half-lives (days)^c^
Benfluralin	335.28	6.5 × 10^−5^ at 25 °C	2.9 × 10^−4^ at 25 °C	-	5.29	9840–11,660	53
Butralin	295.33	1.3 × 10^−5^ at 25 °C	4.9 × 10^−6^ at 25 °C	1 at 25 °C	4.93	3400	180
Dinitramine	322.24	3.6 × 10^−6^ at 25 °C	1.39 × 10^−6^ at 25 °C	1.1 at 25 °C	4.30	4000	30
Ethalfluralin	333.26	8.8 × 10^−5^ at 25 °C	1.3 × 10^−4^ at 25 °C	0.3 at 25 °C	5.11	4100–8400	46
Fluchloralin	355.70	3 × 10^−5^ at 20 °C	1.5 × 10^−5^ at 25 °C	0.9 at 20 °C	5.07	150–550	67
Isopropalin	309.36	1.4 × 10^−5^ at 30 °C	-	0.1 at 25 °C	-	-	-
Nitralin	345.37	9.3 × 10^−9^ at 20 °C 3.3 × 10^−8^ at 30 °C	-	0.6 at 22 °C	-	980	92
Oryzalin	346.36	9.75 × 10^−9^ at 25 °C	1.9 × 10^−9^	2.5 at 25 °C	3.73	600	87
Pendimethalin	281.3	9.4 × 10^−6^ at 25 °C	8.56 × 10^−7^ at 25 °C	pH 4: 0.54 at 20 °C pH 7: 0.33 at 20 °C pH 10: 0.44 at 20 °C	5.2	6500–43,863	174
Profluralin	347.29	6.3 × 10^−5^ at 20 °C	2.9 × 10^−4^ at 25 °C	0.1 at 20 °C	5.58	8600–10,232	110
Trifluralin	335.28	4.58 × 10^−5^ at 25 °C	1.03 × 10^−4^ at 20 °C	pH 5: 18.4 at 25 °C pH 7: 0.221 at 25 °C pH 9: 0.189 at 25 °C	5.34	397–27,900	159

^a^The data comes from PubChem (https://pubchem.ncbi.nlm.nih.gov). Bethesda (MD): National Library of Medicine (US), National Center for Biotechnology Information; 2004. PubChem Compound Summary

^b^No information are available for dinitroanilines listed below, molecular weight in brackets: chlornidine (322.14 g mol^−1^), dipropalin (281.31 g mol^−1^), methalpropalin (347.29 g mol^−1^), nitrofor (307.23 g mol^−1^), prodiamine (350.29 g mol^−1^)

^c^Average value in an aerobic soil

There is little information on the bioaccumulation rate of dinitroanilines in terrestrial and aquatic organisms. However, because there are classified as moderate to highly toxic for fish and aquatic invertebrates (EFSA 2016), it is clearly recommended in label of commercial formulations that no application should be done near/to irrigation canals, or water bodies. Laboratory tests, performed to evaluate sediment and water transfer to aquatic organisms in freshwater habitats, revealed the uptake and bioaccumulation of dinitroanilines in snail *Helisoma* sp., fish *Gambusia affinis*, Cladocera *Daphnia magna* (Kearney et al. 1977; Isensee and Dubey 1983), benthonic oligochaetes *Lumbriculus variegatus* and chironomid larvae *Chironomus riparius* (Mäenpää et al. 2003). In fish, residues tend to accumulate in lipid-rich tissues such as liver, kidneys and muscles (Qiao et al. 2021). Absorption of pendimethalin through dermal exposure was assessed to be depended on moisture and soil organic matter content in the terrestrial amphibian *Bufo americanus* (Van Meter et al. 2016).

The pharmacokinetic profile of pendimethalin was studied in rat females (Osman et al. 2016). The results showed that a single oral dose of active ingredient (109.4 mg kg^−1^ body weight) was absorbed from the intestine and then rapidly distributed in the animal tissues, with concentrations peaking in the serum, liver and kidneys within 12 h and in the brain within 24 h, disappearing after 120 h in the serum and 168 h in the liver, kidneys and brain after administration. In 24 h after the oral administration, pendimethalin was excreted through urine (8.72%) and faeces (14.31%), while the cumulative excretion was of 95.02% in 7 days (Osman et al. 2016). Moreover, biomarkers involved in oxidative stress such as the concentrations of the degradation product of lipid peroxidation, malondialdehyde, and the activities of lactic dehydrogenase, catalase and alkaline phosphatase were significantly enhanced in all the tested tissues suggesting cytotoxic effects. In addition, the distribution, metabolism and excretion of the pendimethalin was studied in rats using (14C)-4-methyl-labelled pendimethalin and (14C)-N-1-ethyl-labelled pendimethalin (Zulalian 1990). The radioactivity was rapidly excreted in urine and faeces and higher residues were found in fat (0.9 ppm), liver and kidney after 96 h. The major metabolic pathways of pendimethalin involved hydroxylation and oxidation of the N-alkyl and 4-methyl groups of the aromatic ring, and the derivate metabolites were found to be predominant in urine (Zulalian 1990). In the crayfish *Procambarus clarkii*, pendimethalin administrated by contact (1.6 mg L^−1^ in water) is rapidly concentrated in the gills (746.5 µg kg^−1^), hepatopancreas (718.26 µg kg^−1^) and muscles (6.07 µg kg^−1^) from 2 h after the initial exposure and declined to undetectable levels by day 21 (Yang et al. 2022). Given that pendimethalin was not released into the water by the animals within the exposure period, a mechanism of metabolic degradation can be hypothesised. Bioaccumulation of trifluralin and pendimethalin was also estimated for 10 up to 12 days in detritivorous earthworm species belonging to the genera *Pheretima* and *Eisenia*. Bioaccumulation factor was higher in *Eisenia* spp. for both tested active ingredients (8.9 for trifluralin and 5.7 for pendimethalin) than *Pheretima* spp. (0.95 for trifluralin and 0.26 for pendimethalin). The uptake and elimination rate differences were related to species-specific variations in the lipid content of the body and the detoxification action of the gut microbiota (Goto and Sudo 2018).

## Acute toxicity

The dinitroanilines resulted toxic to species which have various ecological roles in the food web of aquatic and terrestrial environments. They were classified as slightly to moderately toxic to beneficial terrestrial insects, e.g. 50% mortality in parasitoid wasps depends on the application rate of butralin and pendimethalin (Cheng et al. 2018). The lethal concentration (LC50) of trifluralin is 11 µg bee^−1^ in pollinator *Apis mellifera* (Fernandes et al. 2013) (Table 2). Prodiamine and trifluralin were found to be relatively non-toxic to the detritivorous earthworm *Eisenia fetida* (LC50 > 1000 µg cm^−2^) (Wang et al. 2012). In vertebrates, the lethal dose (LD50) of trifluralin tested by oral exposure has been reported to range from 200 mg kg^−1^ in birds to 10,000 mg kg^−1^ in *Ratus norvegicus* (Fernandes et al. 2013). The oral LD50 is 1250 mg kg^−1^ in male rats, while the dermal LD50 is above 5000 mg kg^−1^ in rabbits, indicating that pendimethalin is slightly toxic by oral exposure and non-toxic by dermal exposure (United State Environmental Protection Agency 1997). Aves can be exposed indirectly to toxic level of dinitroanilines feeding on contaminated fish, arthropods, such as insects and crustaceans, and cultivated grains. Acute dietary risk of exposure has been tested in birds such as mallard duck *Anas platyrhynchos* and bobwhite quail *Colinus virginianus* (Hoffman and Albers 1984; Peterson and Hulting 2004)*.*

**Table 2:** Dinitroaniline acute toxicity values^a^

	Taxa	Benfluralin	Butralin	Dinitramine	Dipropalin	Ethalfluralin	Fluchloralin	Isopropalin	Nitralin	Oryzalin	Pendimethalin	Prodiamine	Profluralin	Trifluralin
**Mammalia**	*Rattus norvegicus*	**LD50** Oral 10 mg/Kg	**LD50** Oral 2500 mg/Kg	**LD50** Oral 3 mg/Kg	**LD50** Oral 3600 mg/Kg	**LD50** Oral 10gm/Kg **LC50** Inhalation 4980 mg/m^3^/4 h	**LD50** Oral 2940 mg/Kg **LC50** Inhalation 8400 mg/m^3^/4 h	**LD50** Oral 5 mg/Kg	**LD50** Oral > 2 gm/Kg **LD50** Skin > 2 mg/Kg	**LD50** Oral 10,000 mg/Kg	**LD50** Oral 1050 mg/Kg **LD50** Intraperitoneal 500 mg/Kg	**LD50** Oral 15,380 mg/Kg **LC50** Inhalation 256 mg/m^3^/4 h **LD50** Skin > 2 gm/Kg	**LD50** Oral 1808 mg/Kg **LD50** Skin > 3170 mg/Kg **LCLo** Inhalation > 3970 mg/m^3^/1 h	**LD50** Oral 1930 mg/Kg **LD50** Skin > 5 mg/Kg **LC50** Inhalation 2800 mg/m^3^/1 h
	*Mus musculus*	**LD50** Oral 5 mg/Kg	**LC50** Inhalation 50 mg/m^3^/4 h	_	_	**LD50** Oral > 10 mg/Kg	**LD50** Oral 730 mg/Kg	**LD50** Oral > 5 mg/Kg	**LD50** Oral > 2 gm/Kg	**LD50** Oral > 10 mg/Kg	**LD50** Oral 1340 mg/Kg **LD50** Intraperitoneal 220 mg/Kg	**LD50** Oral > 15 gm/Kg	_	**LD50** Oral 3197 mg/Kg **LD50** Intraperitoneal 4600 mg/Kg
	*Canis lupus*	**LD50** Oral > 2 mg/Kg	_	_	_	**LD50** Oral > 200 mg/Kg	_	**LDLo** Oral > 2 mg/Kg	_	**LD50** Oral > 1 mg/Kg	**LD50** Oral > 5 mg/Kg	_	_	**LD50** Oral > 10 mg/Kg
	*Felis catus*	_	_	_	_	**LD50** Oral > 200 mg/Kg	_	_	_	**LD50** Oral 1 gm/Kg	_	_	_	_
	*Oryctolagus cuniculus*	**LD50** Oral > 2 mg/Kg **LD50** dermal > 5 g/Kg	**LD50** Skin 200 mg/Kg **LC50** Inhalation 50 g/cu m/4 h	**LD50** skin 2 mg/Kg	_	**LD50** Skin > 2 mg/Kg	**LD50** Skin > 10 mg/Kg	**LDLo** Oral > 2 mg/Kg **LD50** Skin > 2 gm/Kg	**LD50** Skin > 2 mg/Kg	**LD50** Skin > 2 mg/Kg	**LD50** Skin 2260 mg/Kg	_	**LD50** Skin 13,754 mg/Kg **LD50** Percutaneous 3,969 mg/Kg	**LD50** Oral > 2 mg/Kg
	*Meriones unguiculatus*	_	_	_	_	_	_	_	_	**LD50** Oral > 10 gm/Kg	_	_	_	_
**Aves**	*Anas platyrhynchos*	**LD50** Oral > 2 mg/Kg **LOEL** Early life stage 30 weeks > 288 PPM	**LC50** diet > 10,000 PPM/8 days **LOEL** Early life stage 21 weeks > 1500 PPM	**LD50** Oral > 10 mg/Kg	_	**LD50** Oral > 200 mg/Kg **LOEL** Early life stage N.R > 1000 PPM	**LD50** Oral 1300 mg/Kg **LOEL** Early life stage 1 generation > 40 PPM	**LD50** Oral 2 mg/Kg **LC50** 5–10 d 8 d > 5000 PPM	**LC50** 16 d 8 d > 5000 PPM	**LOEL** Early life stage 22 weeks 53 PPM	**LD50** 14 d 8 d 1421 MGK **LOEL** Early life stage 20 weeks 1410 PPM	**LC50** 14 d 8 d > 10,000 PPM **LOEL** Early life stage 1 generation > 1000 PPM	_	**LD50** 3,4 months 8 d > 2000 MGK **LOEL** Early life stage 1 generation 50 PPM
	*Colinus virginianus*	**LD50** Oral > 2 mg/Kg **LC50** > 5000 PPM **LOEL** Early life stage 23 WEEKS 96 PPM	**LD50** > 2250 MGK **LC50** > 10,000 PPM **LOEL** 22 weeks 21 weeks > 1500 PPM	**LD50** Oral > 1200 mg/Kg	_	**LD50** Oral > 200 mg/Kg **LOEL** Early life stage N.R > 1000 PPM	**LD50** Oral 7 mg/Kg **LOEL** Early life stage N.R > 40 PPM	**LD50** Oral 1 gm/Kg **LC50** 10–14 d 8 d > 5000 PPM	**LD50** 21 weeks 14 d 506.7 MGK **LC50** 11 d 8 d > 5000 PPM	**LOEL** Early life stage 1 generation > 1000 PPM **LOEL** Early life stage 22 weeks 311 PPM	**LC50** 10-14d 8 d 4187 PPM **LOEL** Early life stage 20 weeks > 1410 PPM	**LD50** 39 weeks 14 D > 2250 MGK **LC50** 14 d 8 d > 10,000 PPM **LOEL** 20 weeks N.R > 1000 PPM	_	**LD50** 16 weeks 14 d > 2000 MGK **LOEL** Early life stage 1 generation > 50 PPM
	*Coturnix japonica*	_	_	_	_	**LD50** 8 weekss 14 d > 2000 MGK	_	_	_	*_*	_	_	_	_
	*Gallus domesticus*	**LD50** Oral > 2 mg/Kg	_	_	_	_	_	**LD50** Oral 2 mg/Kg	_	**LD50** Oral 1 mg/Kg	_	_	_	**LD50** Oral > 2 mg/Kg
	*Phasianus colchicus*	_	_	_	_	_	_	_	_	_	_	_	_	**LD50** 3 months 14 D > 2000 MGK
	*Serinus canaria*	_	**LD50** Adult 14 d > 2000 (regurge) MGK	_	_	_	_	_	_	**LD50** Adult 14 d > 1200 (regurge) MGK	**LD50** 28 Weeks 14 d > 2000 limit test MGK	**LD50** Adult 14 d > 2600 limit test MGK	_	_
	*Taeniopygia guttata*	_	_	_	_	_	_	_	_	_	_	_	_	**LD50** Adult 14 D > 2000 MGK
**Amphibia**	*Anaxyrus fowleri*	_	_	_	_	_	_	_	_	_	_	_	_	**LC50** 96 h 0.10 PPM
**Fish**	*Carassius auratus*	**LC50** 96 h 0.8 mg/L	_	_	_	**LC50** 1.2 g 96 h 260 PPB	_	_	_	_	_	_	_	**LC50** 1.0 g 96 h 145 PPB
	*Cyprinodon variegatus*	**LC50** 0.2 g 96 h > 29 PPB **LC50** 0.2 g 96 h 29 PPB	**LC50** 25 mm 96 h > 0.18 PPM **LOEC** Early life stage 34 d 0.072 PPM	_	_	**LC50** N.R. 96 h 240 PPB **LOEC** Early life stage 33 d 2.7 PPB	_	_	_	**LC50** 0.33 g 96 h > 3.04 PPM **LOEC** Early life stage 34 d 180 PPB	**LOEC** Early life stage 34 D 23 PPB	**LC50** 0.24 g 96 h > 450 PPB	_	**LC50** Juvenile 96 h 160 PPB **LOEC** Early life stage N.R 4.8 PPB
	*Ictalurus punctatus*	_	_	_	_	_	**LC50** 0.6 g 96 h 120 PPB	_	_	_	**LC50** 1.5 g 96 h 418 PPM	**LC50** N.R 96 h > 52 PPM	_	**LC50** 5.2 g 96 h 210 PPB **LOEC** Full life cycle 61 weeks 5.1 PPB
	*Lepomis macrochirus*	**LC50** Juvenile 96 h > 45 PPB	**LC50** 0.2 g 96 h 1.0 PPM	**LC50** 0.27 g 96 h 5.7 PPM	_	**LC50** 1.2 g 96 h 101.9 PPB	**LC50** 0.5-3 g 96 h 16.0 PPB	**LC50** Juvenile 96 h 24,300 PPB	_	**LC50** N.R 96 h 2.88 PPM	**LC50** 0.9 g 96 h 90.4 PPB	**LC50** 0.72 g 96 h > 552 PPB	_	**LC50** 0.9 g 96 h 8.4 PPB
	*Micropterus salmoides*	_	_	_	_	_	_	_	_	_	_	_	_	**LC50** 0.7 g 96 h 75 PPB
	*Oncorhynchus mykiss*	**LC50** 1.1 g 96 h > 48 PPB **LOEC** Early life stage 49 d 5.0 PPB	**LC50** 0.33 g 96 h 0.37 PPM	_	_	**LC50** 0.5-3 g 96 h 9.6 PPB **LOEC** Early life stage 50 d 1.4 PPB	**LC50** 0.5-3 g 96 h 12.0 PPB	**LC50** Juvenile 96 h 803 PPB	_	**LC50** N.R 96 h 3.26 PPM **LOEC** Early life stage 66 d > 0.46 PPM	**LC50** 1.3 g 96 h 86.6 PPB	**LC50** 1.1 g 96 h > 829 PPB **LOEC** Early life stage 87 d 25 PPB	_	**LC50** 0.8 g 96 h 10 PPB **LOEC** Early life stage 48 d 4.32 PPB
	*Pimephales promelas*	**LC50** 0.9 g 96 h > 100 PPB	**LOEC** Early life stage 32 d 0.016 PPM	_	_	_	**LOEC** Early life stage 12 months > 9 PPB	_	_	**LOEC** Early life stage 34 d 0.43 PPM	**LOEC** Full life cycle 288 d 9.4 PPB	**LC50** 0.29 g 96 h > 26 limit test PPB **LOEC** Full life cycle 154 d 3.8 PPB	_	**LC50** 0.8 g 96 h 105 PPB **LOEC** Early life stage 35 d 0.7 PPB
	*Stizostedion vitreum*	_	_	_	_	_	_	_	_	_	_	_	_	**LC50** 1.4 g 24 h 180 PPB
**Crustacea**	*Americamysis bahia*	**LC50** 24 h 96 h 43 PPM **LOEC** Full life cycle 28 d 4.3 PPB	**LC50** < 24 h 96 h 0.070 PPM **LOEC** Full life cycle 28 d 0.074 PPM	_	_	**LC50** N.R 96 h 230 PPB **LOEC** Full life cycle 28 d 57.8 PPB	_	_	_	**LOEC** Full life cycle 29 d 26.6 PPB	**LOEC** Full life cycle 28 d 1.6 PPB	**LC50** 24 h 96 h > 310 PPB **LOEC** Full life cycle 28 d 20 PPB	_	**LOEC** Full life cycle 28 d 2.61 PPB
	Asellus brevicaudus	_	_	_	_	_	_	_	_	_	_	_	_	**EC50** Juvenile 96 h > 1000 PPB
	*Cancer magister*	_	_	_	_	_	_	_	_	_	_	_	_	**LC50** N.R 96 h 330 PPB
	*Daphnia magna*	**EC50** 1st-I 48 h 94.8 PPB **LOEC** Full life cycle 21 d 30.8 PPB	**EC50** < 24 h 48 h 1.0 PPM **LOEC** Full life cycle 21 d 150 PPM	**EC50** 24 h 48 h 1.3 PPM	_	**EC50** < 24 h 48 h 18.1 PPB **LOEC** Full life cycle 21 d 37.4 PPB	**EC50** < 24 h 48 h 560.0 PPB	**EC50** 1st-I 48 h 269 PPB	_	**EC50** N.R 48 h 1.45 PPM **LOEC** Full life cycle 21 d 0.608 PPM	**EC50** 1st-I 48 h 280 PPB **LOEC** Full life cycle 21 d 17.2 PPB	**EC50** < 24 h 48 h > 83 PPB **LOEC** Full life cycle 21 d 2.6 PPB	_	**EC50** 1st-I 48–96 560 PPB **LOEC** Full life cycle 64 d 7.2 PPB
	*Daphnia pulex*													**EC50** 1st-I 48 h 625 PPB
	*Gammarus* spp.	*G. fasciatus* **LC50** (Scud, mature) 1.1 mg/L/96 h **EC50** Juvenile 48 h 4000 PPB	_	_	_	_	*G. pseudolimnaeus* **LC50** Juvenile 96 h 56 PPB	_	_	_	_	_	_	*G. fasciatus* **LC50** Adult 96 h 2200 PPB
	*Hyalella Azteca*	**LOEC** Full life cycle 42 d > 78 (porewater) PPB	**LOEC** Full life cycle 42 d > 0.17 (porewater) PPM	_	_	**LC50** 7 d 10 d > 0.24 (porewater) PPM **LOEC** 7 d 10 d 0.14 (porewater) PPM	_	_	_	**LC50** Early life stage 10 d 9790 (overwater) PPB **LC50** Early life stage 10 d 231 (sediment) MGK **LOEC** Full life cycle 42 d > 75.3 (porewater) PPM	**LOEC** Early life stage 42 d 1.8 (overwater) PPB	**LOEC** Early life stage 42 d > 210 (porewater) PPB	_	**LOEC** Full life cycle 30 d 18.9 (porewater) PPM **LOEC** 871 (sediment) MGK
	*Lepomis macrochirus*	_	_	_	_	**LC50** 0.5–3 g 96 h 17 PPB	_	_	_	_	_	_	_	_
	*Leptocheirus plumulosus*	**LOEC** Juvenile 10 d > 19 (porewater) PPB	_	_	_	**LC50** 10 d 10 d > 46 (porewater) PPB	_	_	_	_	**LOEC** Early life stage 10 d > 1.3 (overwater) PPB	**LC50** Juvenile 10 d > 51 (porewater) PPB	_	**LC50** Juvenile 10 d > 0.22 (porewater) PPM
	*Palaemonetes pugio*	_	_	_	_	_	_	_	_	**LC50** 0.06 g 96 h > 3.11 PPM	_	_	_	**LC50** Juvenile 96 h 638.5 PPB
	*Penaeus duorarum*	_	_	_	_	_	_	_	_	_	**LC50** 1.4 g 96 h 1600 PPB	_	_	_
	*Palaemonetes kadiakensis*	_	_	_	_	_	_	_	_	_	_	_	_	**LC50** Juvenile 96 h 37 PPB
	*Procambarus simulans*	_	_	_	_	_	_	_	_	_	**LC50** 2.4 g 96 h > 1000 PPB	_	_	_
	*Simocephalus serrulatus*	_	_	_	_	_	_	_	_	_	_	_	_	**EC50** 1st-I 48 h 900 PPB
**Mollusca**	*Crassostrea virginica*	**EC50** SPAT 96 h > 30 PPB	**EC50** SPAT 96 h 0.12 PPM	_	_	**EC50** SPAT 96 h 102 PPB	_	_	_	**EC50** SPAT 96 h 0.285 PPM	**EC50** Embryo larval 48 h 210 PPB	**EC50** SPAT 96 h 370 PPB	_	**EC50** SPAT 96 h > 110 PPB
	*Mytilus edulis*	_	_	_	_	_	_	_	_	_	_	_	_	**EC50** Embryo larval 48 h > 120 PPB **LOEC** SPAT 48 h 20 PPB
**Anellida**	*Eisenia fetida*	_	_	_	_	_	_	_	_	_	_	_	_	**LC50** 0.36 g 14 d > 1000 soil MGK
**Insecta**	*Apis mellifera*	**LD50** Worker 48 h > 14.5 µg/BEE	**LD50** Adult 48 h 92.8 µg/BEE	_	_	**LD50** Worker 48 h 51 µg/BEE **LD50** Larvae 72 h 73 µg/ai/larva **LC50** Larvae 72 h 2098 in diet MGK	**LD50** Worker 48 h > 90.6 µg/BEE	_	_	**LD50** Adult 48 h > 11 µg/BEE	**LD50** Adult 48 h > 49.8 µg/BEE	**LD50** Worker 48 h > 100 µg/BEE	_	**LD50** Adult 48 h > 24.17 µg/BEE
	*Chironomus* spp.	_	*C. dilutes* **LOEC** Full life cycle 63 d > 0.16 (porewater) PPM	_	_	_	*C. plumonthsus* **LC50** 3rd In 96 h 5.6 PPB	_	_	*C. tentans* **LC50** 10 d 10 d > 362 (overwater) PPB **LOEC** Full life cycle 47 d 3.4 (porewater) PPM	*C. riparius* **EC50** < 2 d 28 d > 203 (sediment) MGK *C. dilutes* **LOEC** Full life cycle 63 d 0.12 (overwater) PPB	*C. tentans* **LOEC** Full life cycle 62 d 3.5 (overwater) PPB **LOEC** 35.8 sediment MGK	_	*C. riparius* **EC50** Early life stage 28 d 6900 (overwater) PPB **LOEC** Early life stage 28 d 500 (overwater) PPB **LOEC** Early life stage 21 d > 19.9 (porewater) PPM
	*Pteronarcys californica*	_	_	_	_	_	_	_	_	_	_	_	_	**LC50** YC 2 96 h 2800 PPB

The data indicates toxicity test, age of the samples (days, weight, stage), length of the test, dose and unit respectively

^a^The data comes from PubChem (https://pubchem.ncbi.nlm.nih.gov) PubChem Compound Summary; EPA Pesticide Ecotoxicity Database https://ecotox.ipmcenters.org/

*MGK*, milligrams/Kg body weight; *YC 2*, second year class

Acute toxicity to aquatic organisms is moderate to high. Omnivorous and carnivorous freshwater fish from riparian and stream zones have been tested to evaluate the risk exposure from runoff of herbicides applied in surrounding agricultural areas of rivers, lakes and ponds (Table 2). The LC50 value after 96-h exposure to pendimethalin was very similar in *Clarias batrachus* (3.55 mg L^−1^)(Gupta and Verma 2022), *Channa punctatus* (3.6 mg L^−1^) (Ahmad and Ahmad 2016) and Nile tilapia (3.55 mg L^−1^)(Ahmed and Moustafa 2010), but very different from the values obtained for bluegill sunfish (0.19 mg L^−1^) and in rainbow trout (0.138 mg L^−1^) (United State Environmental Protection Agency 1997). In invertebrates, the LC50 of trifluralin is 0.56 mg L^−1^ in *Daphnia magna* and 12 mg L^−1^ in the red swamp crayfish *Procambarus clarkii*, while the LC50 in fish ranges from 10 µg L^−1^ to 0.21 mg L^−1^ (Fernandes et al. 2013). The LC50 of trifluralin, ethalfluralin and isopropalin ranges from 63 to 150 mg L^−1^ in Cladocera (Sanchez-Bayo 2006). The acute toxicity of pendimethalin was studied in *Daphnia magna* (LC50 = 400 µg L^−1^), oysters (LC50 = 210 µg L^−1^), penaeid shrimps (LC50 = 1600 µg L^−1^) and various fish species (LC50 ranging from 137 µg L^−1^ in Cyprinidae to 890 µg L^−1^ in rainbow trout) (Fliedner 1997; Vighi et al. 2017). In the freshwater snail *Biomphalaria alexandrina*, the LC50 were 5.560 mg L^−1^ for butralin and 2.148 mg L^−1^ for pendimethalin (Ibrahim et al. 2019).

In addition, other reliable toxicological tests, for evaluating acute risks to aquatic and terrestrial species, such as the half maximum effective concentration (EC50) and the sub-chronic lowest observed effect concentration (LOEC), as well as the LC50, given in the PubChem datasets, are summarised in Table 2. Further information on the potential health hazards of dinitroanilines can also be found in the registration reports of the EFSA (EFSA 2016; EFSA et al. 2019) and Environmental Protection Agency (EPA) of the USA (United State Environmental Protection Agency 1997) active substance database. It should be noted that despite the limited information on the toxic effects on humans, EFSA has set the maximum residue levels for dinitroanilines (from 0.01 to 0.7 mg kg^−1^ for different active ingredients) that are admitted in food as a precautionary measure (Reg. EU 2019/1791 and 2018/687).

## Side effects of chronic exposure

**Fig. 1:**
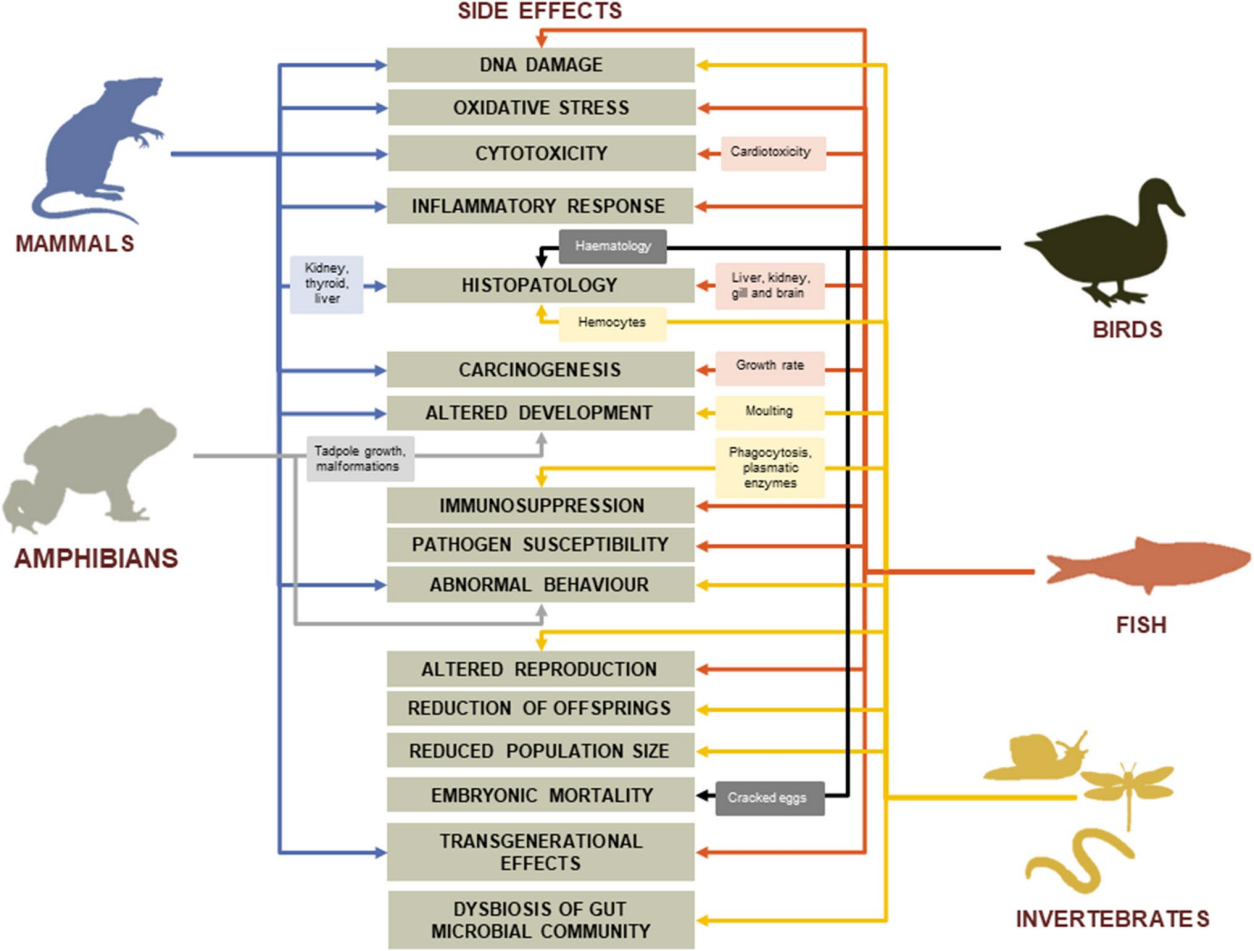
Schematic diagram of side effects across taxa

Despite the high degree of sequence conservation for all tubulins in organisms (Dutcher 2001; Janke and Magiera 2020), there is little knowledge about the chronic toxicological action of this herbicide class on animal cells. A low affinity for the cell’s tubulin is currently indicated in animals (Blume et al. 2003; Morrissette and Sept 2009; Rose et al. 2016). However, pendimethalin has been tested to bind the calf thymus cell DNA preferentially to the G–C-rich sequences (Ahmad et al. 2016) and serum albumin of bovine, pigs, sheep, rabbits (Lee et al. 2019) and human (Ahmad et al. 2020). Low-doses or sublethal exposure to dinitroanilines have shown variable side effects across taxa at all levels of biological organisation (Fig. 1, Tables 3 and 4), as described below.

**Table 3:** Dinitroaniline sublethal effects on invertebrates at different biological levels

Dinitroaniline	Molecular	Cells and organs	Behaviour	Physiology	Population	Community
Benfluralin				*Octodrilus complanatus*; reduction of growth and survival; 675, 1350 and 2700 g ha^−1^ (Travlos et al., 2017)		
Butralin	*Biomphalaria alexandrina*; transaminase activities and total albumin level in the haemolymph; 2417 (LC_10_) and 3906 (LC_25_) mg L^−1^ (Ibrahim et al. 2019)	*Biomphalaria alexandrina*; increased phagocytic activity of haemocytes; 0.556 (LC_0_), 2.41 (LC_10_), 3.906 (LC_25_) mg L^−1^ (Ibrahim et al. 2019)				
Oryzalin		*Caenorhabditis elegans*; altered meiotic oocyte nuclei and chromosome; 25 μM (Goldstein 2021)		*Caenorhabditis elegans*; altered reproduction; 25 μM (Goldstein 2021) *Tiphia vernalis*; increased mortality; 0.24 kg ha^−1^ (Oliver et al. 2009)		
Pendimethalin	*Biomphalaria alexandrina*; transaminase activities and total albumin level in the haemolymph; 0.535 (LC_10_) and 1299 (LC_25_) mg L^−1^ (Ibrahim et al. 2019) *Harpalus (Pseudoophonus) rufipes* reduction of phenoloxidase and lysozyme like enzymes; 4 L ha^−1^; (Giglio et al. 2019)	*Biomphalaria alexandrina*; increased phagocytosis activities of haemocytes; 0.214 (LC_0_), 0.535 (LC_10_), 1.299 (LC_25_) mg L^−1^ (Ibrahim et al. 2019) *Harpalus (Pseudoophonus) rufipes*; reduced density and phagocytic activity, altered haemocyte formula and morphology in circulating cells; 4 L ha^−1^ (Vommaro et al. 2021)	*Eisenia fetida*; attraction; 0.316 and 1 mg kg^−1^ soil; avoidance; 3.16 mg kg^−1^ soil (Khunteta and Singh 2014)	Root-knot nematodes; slowed growth; 3 µL cm^−2^ (Das et al. 1998; Dopierala and Giebel 2002) *Folsomia candida*; reduction of offsprings; 1 m kg^−1^; 4 kg ha^−1^ *Eisenia fetida*; lower weight and growth; LOEC 10 mg kg^−1^ (Belden et al., 2005) *Cyphoderus javanus*; alteration in moulting, hatching and sexual maturity; 72.6 g ha^−1^(1/8 LC_50_) (Chakravorty et al. 2015) *Xenylla whelchi*; alteration in moulting, hatching and sexual maturity; 23.8 g ha^−1^(1/8 LC_50_) (Haque et al. 2011) *Tiphia vernalis*; increased mortality; 1.68 kg ha^−1^ (Oliver et al. 2009)	Root-knot nematodes; reduced population size; 3 µL cm^−2^ (Das et al. 1998; Dopierala and Giebel 2002)	*Pterostichus melas italicus*; dysbiosis gut microbial communities; 4 L ha^−1^ (Giglio et al. 2021)
Trifluralin	*Drosophila melanogaster*; genetic changes somatic cells of the wing’s imaginal discs; 10 mM (Kaya et al. 2004)					

Data indicates in the columns: model species, side effects and doses, respectively

**Table 4:** Dinitroaniline sublethal effects on vertebrates at different biological levels

Dinitroaniline	Molecular	Cells and organs	Behaviour	Physiology	Population	Community
Benfluralin		Rat and human’s thyroid rodent hepatocytes; follicular adenoma and carcinoma; ≥ 2.5 mg L^−1^ (Strupp et al. 2020b, 2020a)		*Xenopus laevis*; Altered tadpole development; 0.004, 0.020, 0.10 mg L^−1^ water (Strupp et al. 2020b)		
Butralin		Rat kidney tissues; oxidative stress, decreased antioxidant enzymes activities, elevated DNA damage, and histological changes; acceptable daily intake 0.5 mg kg^−1^ (Refaie et al., 2021)				
Dinitramine		*Danio rerio* embrio; cardiotoxicity, inflammatory response, apoptosis; 1.6, 3.2, and 6.4 mg L^−1^ (Park et al. 2021)		*Danio rerio*; embryonic growth and survival; 1.6, 3.2, and 6.4 mg L^−1^ (Park et al. 2021)		
Fluchloralin			Mouse; Activity alteration; 200 mg kg^−1^ (Mitchell et al. 1989)			
Pendimethalin	Chinese hamster lung fibroblasts (V79) and human lymphocytes; oxidative stress pathway and chromosomal damage; 66 µM (Kılıç et al. 2018) *Channa punctatus*; decreased protein content in liver, testes, ovary; 0.660 mg L^−1^; (Kalita et al., 2018) Human lung cells (A549); altered expression level of carcinoma–related genes; 100 µM (Sarigöl-Kiliç and Ündeğer-Bucurgat, 2018)	ovary (CHO) cells; cytotoxicity; 0.1–100 µM (Patel et al. 2007) Rat thyroid derived cell line (FRTL-5 cells); Cytotoxicity, reduced synthesis and secretion of thyroglobulin; 5 ng µL^−1^ (Pan et al. 2004) hepatocyte mitochondria of rats; enhanced oxidative phosphorylation, decreased membrane potential; 23–154 mg mL^−1^ commercial formulation (Četkauskaitė et al., 2006) Hepatocytes mitochondria of rats; active ingredient 100 and 1000 µM (Yamano and Morita, 1995) Human lymphocytes and rat bone-marrow cells; oxidative stress, apoptosis; 200 μM (Ansari et al. 2018) Mouse bone marrow; chromosomal aberrations and micronuclei; 0.05 and 0.1 mL commercial formulation (Dimitrov et al. 2006) Rat’s liver, renal and cardiac tissues; histological alterations; 50 mg kg^−1^ body weight per day (oral) (Ansari et al. 2018) *Danio rerio*; spinal curvature, yolk sac and pericardial edema; 703.3 and 1406.6 µg L^−1^ (Wang et al. 2022) *Oncorhynchus mykiss*; aberrant expression of immune genes during infection; 200 ng L^−1^ per day (Dupuy et al., 2019) *Channa punctatus*; histopathological changes in liver, kidney, gill and brain; 0.0005 and 0.0008 mg L^−1^ (Tabassum et al., 2015 and 2016) *Channa punctatus*; genotoxic and oxidative effects in gills, erythrocytes and liver; 0.9, 1.8, and 2.7 mg L^−1^ (Ahmad and Ahmad 2016) *Cyprinus carpio*; erythrocyte and hemopoietic tissue alterations; 2.5 µg L^−1^ (Bojarski et al. 2018; Lutnicka et al. 2018) *Oreochromis niloticus*; Genotoxic effects in the erythrocytes; 0.52 mg L^−1^ (Hamed and El-Sayed 2019)		*Danio rerio;* Mortality; 0.5 mg L^−1^ and 0.05 mg L^−1^ (Merola et al. 2022) *Oncorhynchus mykiss;* leukopenia oxidative stress and increase of the bile secretion; 500 ng L^−1^, 800 ng L^−1^ (Danion et al. 2012 and 2014) *Clarias batrachus;* endocrine disruptor and reproduction dysfunction; 1/20 LC_50_, 1/15 LC_50_, 1/10 LC_50_ (Gupta and Verma 2020, 2020b)	*Danio rerio;* reproductive dysfunction in the F1 generation, transgenerational effect on development in the F2; 0.5, 5 and 50 μg L^−1^ (Xie et al. 2019) *Oncorhynchus mykiss;* transgenerational effect on larval development; 0.85 μg L^−1^ (Danion et al. 2018) Farmers and nursery workers; lung cancer risk; field rate (Alavanja et al. 2004; Hou et al. 2006)	Oncorhynchus mykiss; pathogen susceptibility; 0.85 μg L^−1^ (Danion et al. 2018)
Trifluralin	Chinese hamster lung fibroblasts (V79) and human lymphocytes; oxidative stress pathway and chromosomal damage; 128 µM (Kılıç et al. 2018) Human lung cells (A549); altered expression level of genes carcinoma-related; 100 and 500 µM (Sarigöl-Kiliç and Ündeğer-Bucurgat 2018)	Human peripheral blood lymphocytes; cytotoxic and genotoxic effects; 50 µg mL^−1^ (Ribas et al. 1996) *Oreochromis niloticus*; genotoxic effects in the erythrocytes; 1 µg L^−1^ (Könen and Çavaş, 2008)		Rats and rabbits; loss fetal body weights and viability; 100 and 250 mg kg^−1^ day^−1^ (Byrd et al. 1995)	*Anas platyrhynchos* *Colinus virginianus*; increased cracked eggs; 1.79 g L^−1^ (Hoffman and Albers 1984; Peterson and Hulting 2004)	

Data indicated in the columns: tested species, side effects and doses, respectively

### *Genotoxicity and cytotoxicity*

All in vitro analyses on vertebrate’s cells revealed cytotoxic and genotoxic effects of exposure to dinitroanilines for both the active ingredients and the commercial formulates in both vertebrates and invertebrates (Fig. 1). Activation of the oxidative stress pathway and chromosomal damage were assessed in Chinese hamster lung fibroblasts (V79) and lymphocytes exposed to pendimethalin (66 µM) and trifluralin (128 µM) (Kılıç et al. 2018). In ovarian cells (CHO) exposed to pendimethalin, a significant decrease in cell viability as measured by metabolic activity of mitochondria and an increase in DNA damage were observed at 0.1, 1, 10 and 100 µM after 3 h (Patel et al. 2007). Pendimethalin had cytotoxic effects on rat thyroid–derived cell line (FRTL-5 cells) at the concentration of 5 ng µL^−1^ interfering with the synthesis and secretion of thyroglobulin (Pan et al. 2004). The commercial formulation (Stomp) of this herbicide enhanced the oxidative phosphorylation pathway in a concentration-dependent manner and significantly decreased membrane potential in liver mitochondria of rats (Četkauskaitė et al. 2006). Similar cytotoxic findings were also reported for the application of the technical active ingredient (98% purity) at 100 and 1000 µM (Yamano and Morita 1995). Pendimethalin at a concentration of 200 μM induces oxidative stress in vitro and triggered apoptosis in human lymphocytes and rat bone marrow cells (Ansari et al. 2018). Trifluralin, profluralin and fluchloralin have been found to be metabolised in vitro by rat liver microsomes (Nelson et al. 1977). However, no further information is available on the detoxification pathway or their mode of action on animal cells. In vivo exposure to butralin for 30 days induced oxidative stress and decreased antioxidant enzyme activities, and it increased DNA damage and histological changes in rat kidney tissue (acceptable daily intake 0.5 mg kg^−1^) (Refaie et al. 2021). In the freshwater snail *B. alexandrina*, sublethal doses of a commercial butralin-based formulation (Amex 48% EC; LC25 = 3.906 mg L^−1^ for 48 h) cause DNA strand breaks and loss of viability and phagocytic activity of circulating haemocytes (Ibrahim et al. 2019). Sister-chromatid exchanges, chromosomal aberrations and micronuclei showed that trifluralin is capable of exerting weak cytotoxic and genotoxic effects in cultured human peripheral blood lymphocytes (Ribas et al. 1996). Oral exposure to 0.05 and 0.1 mL of a commercial pendimethalin-based formulation (Stomp) significantly increased the frequency of chromosomal aberrations and micronuclei in mouse bone marrow polychromatic erythrocytes (Dimitrov et al. 2006).

Recent overviews of the registration documents suggest that the liver, thyroid and kidney are the most common in vivo target organs for dinitroaniline compounds such as benfluralin, butralin, ethafluralin, oryzalin, pendimethalin, prodiamine and trifluralin (Ahmad et al. 2018; Leonard et al. 2019; Strupp et al. 2020b). Benfluralin increased the incidence of thyroid follicular adenoma and carcinoma at high dietary concentrations (≥ 2500 ppm) in rats and humans (Strupp et al. 2020b) and in rodent hepatocytes (Strupp et al. 2020a). Genotoxic effects were recorded in erythrocytes of Nile tilapia, *Oreochromis niloticu*s exposed to pendimethalin (Hamed and El-Sayed 2019) and both the active ingredient and the commercial formulation (Treflan, a.i. 480 g L^−1^) of trifluralin (Könen and Çavaş 2008). The expression levels of P53, BAX, BCL-2, CAS3, CAS9, BIRC and PPIA (housekeeping) genes associated to apoptosis were altered in A549 human lung carcinoma cells after exposure in vitro to pendimethalin and trifluralin (Sarigöl-Kiliç and Ündeğer-Bucurgat 2018).

### *Morphological, physiological and behavioural alterations*

In organisms exposed to dinitroanilines, morphological and functional alterations have been found to have adverse effect on growth, longevity, reproduction and behaviour of individuals that survived the initial exposure (Fig. 1, Tables 3 and 4). In invertebrates, oryzalin at a concentration of 25 μM caused severe morphological and ultrastructural changes in meiotic oocyte nuclei and chromosomes of the free-living nematode *Caenorhabditis elegans* affecting the reproduction (Goldstein 2021). The earthworm *Octodrilus complanatus* exposed to the recommended field doses of benfluralin (675, 1350 and 2700 g of the active ingredient per ha) showed a dose–response trend for reduction in growth and survival rates from 1 to 5 weeks after treatment (Travlos et al. 2017)*.* In the embryonic stage of *Drosophila melanogaster*, oral exposure to trifluralin (10 mM) was studied for genetic changes induced in somatic cells of wing imaginal discs (Kaya et al. 2004). Exposure to sublethal doses of dinitroanilines has effects on physiological parameters of organisms by activating detoxification mechanisms or interfering with cellular activities involved in pathogen clearance, making organisms more susceptible to infection. Commercial formulations of butralin (Amex 48% EC; LC10 = 2.417 and LC25 = 3.906 mg L^−1^) and pendimethalin (Stomp 50% EC; LC10 = 0.535 and LC25 = 1.299 mg L^−1^) caused a concentration-dependent change in biochemical parameters such as transaminase activities and total albumin levels in the haemolymph of the freshwater snail *B. alexandrina*, which is likely related to antioxidant processes activates to maintain homeostasis (Ibrahim et al. 2019). Moreover, this study has highlighted that both herbicides have genotoxic and cytotoxic effects on circulating haemocytes affecting viability and increasing phagocytic activities.

Laboratory exposure to a field dose (1320 gr ha^−1^) of a commercial pendimethalin-based formulation (Activus, a.i. 330 g L^−1^) was found to interfere with the cellular and humoral immune responses of the carabid beetle *Harpalus (Pseudoophonus) rufipes* (Giglio et al. 2019; Vommaro et al. 2021) and cause DNA damage in samples collected from treated fields (Cavaliere et al. 2019). In particular, the decrease in circulating haemocytes and the alteration in the haemogram observed in this species were considered to be due to an inflammatory process involving haemocytes in the repair of damaged tissues. The decrease in phagocytosis index after 48 h of exposure may be due to the ultrastructure modification observed in the haemocytes involved in this cell-mediated immune response. In addition, these studies have shown that exposure to pendimethalin has effects on humoral parameters such as plasmatic phenoloxidase and lysozyme-like enzyme levels.

In vertebrates, histological alterations were observed in liver, kidney and cardiac tissues of rats after oral exposure to 50 mg kg^−1^ body weight per day of pendimethalin (Ansari et al. 2018). Studies performed to evaluate potential toxicity on development highlighted loss of foetal body weight and viability in rats and rabbits exposed to trifluralin (475 and 500 mg kg^−1^ respectively) (Byrd et al. 1995) and in the tadpole development of the amphibian *Xenopus laevis* exposed to benfluralin (0.004, 0.020 and 0.100 mg L^−1^ of a.i. in water) (Strupp et al. 2020b).

The zebrafish *Danio rerio* and the rainbow trout are the main models used to test the toxicological effects of dinitroanilines in aquatic environments. Exposure to 1.6, 3.2 and 6.4 mg L^−1^ of dinitramine induced developmental malformation in zebrafish embryos, caused cardiotoxicity in larvae and triggered an inflammatory response and apoptotic cell death, impairing embryonic growth and survival (Park et al. 2021). Zebrafish larvae exposed to pendimethalin (0.5 mg L^−1^ and 0.05 mg L^−1^) exhibited mortality and developed sublethal alterations, including biochemical changes in antioxidant enzymes (Merola et al. 2022). Moreover, morphological modifications, including spinal curvature, yolk sac and pericardial oedema, occurred in zebrafish embryos exposed to 0.7 and 1.4 mg L^−1^ of pendimethalin, as well as alterations in genes involved in the mitochondrial electron transport chain (Wang et al. 2022), while reduced heart rate, survival rate and body length were observed at 3, 4 and 5 mg L^−1^.(Meng et al. 2021). Other adverse effects observed in adult rainbow trout due to exposure to a commercial formulation based on pendimethalin include aberrant expression of immune genes during infection (200 ngL^−1^ per day) (Dupuy et al. 2019), oxidative stress and an increase in the bile secretion (500ngL^−1^, 800ngL^−1^) (Danion et al. 2014), and changes in haematological (leukopenia) and immunological parameters (Danion et al. 2012). This herbicide at sublethal concentrations of 0.5 and 0.8 µg L^−1^ induced biochemical and histopathological changes in the liver, kidney, gills (Tabassum et al. 2016) and brain (Tabassum et al. 2015) of the freshwater fish *Channa punctatus* and reduced protein content in the liver, testes and ovary of the spotted snakehead (Kalita et al. 2018). Moreover, genotoxic and oxidative side effects were recorded to be concentration- and time-dependent in the gills, erythrocytes and liver at sublethal concentrations of 0.9, 1.8 and 2.7 mg L^−1^, respectively (Ahmad and Ahmad 2016). Pendimethalin exposure induces alterations in erythrocytes and hematopoietic tissues in common carp (*Cyprinus carpio*) (Bojarski et al. 2018; Lutnicka et al. 2018) and histological changes in gill epithelium and general branchial functions of *Oreochromis (Tilapia) nilotica* (Abd-Algadir et al. 2011). Different concentrations of the pendimethalin-based commercial formulation Stomp were tested on adults of *O. niloticus*. Specimens exposed to 5% of the 96 h-LC50 dose showed changes in growth, biochemical parameters and DNA damage in liver and gill tissues (El-Sharkawy et al. 2011), while intraperitoneally injected single doses of 5.08, 2.54 and 1.02 mg kg^−1^ (corresponding to 1/5, 1/10 and 1/25 of the LD50) caused a decrease in mitochondrial cytochrome content and histopathological changes in gills, liver and brain (Nassar et al. 2021). Moreover, leukocytosis, hyperglobulinemia, hyperglycemia and oxidative stress were observed after exposure to 50% of the 96-h LC50 (2.5 mg/L) over a 4-day period (El-Sayed et al. 2015). In the Nile catfish *Clarias gariepinus*, the same dose administrated for 15 days resulted in a decrease in phagocytic activity, an increase in oxidative stress and necrosis of liver tissue (Moustafa et al. 2016), while the commercial formulation Vestaline altered the immunological parameters of the juvenile stage exposed for 8 weeks (Odo 2019).

In males and females of freshwater air-breathing catfish *Clarias batrachus*, several biomarkers, including sex steroid levels, plasma vitellogenin concentrations and gonadal aromatase activity, suggest that pendimethalin may act as an endocrine disruptor and cause reproductive disorders (Gupta and Verma 2020). Oral exposure also impaired estrogenic activity in immature female rats and resulted in an increase of uterine weight at the doses of 300 mg/kg/day and 600 mg/kg/day suggesting an interference on hypothalamo-pituitary–gonadal axis (Ündeǧer et al. 2010).

Behavioural tests showed changes in activity in mice exposed to a fluchloralin-based commercial formulation (Basalin 45%) (Mitchell et al. 1989). The pendimethalin concentration of 281.3µL^−1^ is sufficient to reduce locomotory activity of zebrafish larval stage in a week (Wang et al. 2022). The earthworm *E. fetida* was attracted to pendimethalin at low concentrations of 0.316 and 1 mg kg^−1^ of dry soil and showed higher average avoidance at concentrations of 3.16 mg kg^−1^ or greater (Khunteta and Singh 2014). However, there are no studies on the direct effects of these herbicides on animal behaviour, such as foraging and space use.

### *Sublethal effects at population and community level*

Few studies have addressed the potential effects of cumulative use and continuous exposure to dinitroanilines on population and community structure. In invertebrates, a reduction in population and growth rate was observed in numerous organisms (Fig. 1, Tables 3 and 4), such as the root-knot nematodes exposed to recommended field doses of pendimethalin (3 µL cm^−2^) (Das et al. 1998; Dopierala and Giebel 2002). In contrast, it had no significant effects on larval survival and pupation and eclosion rates of *Apis mellifera ligustica* and *A. cerana cerana* at doses of 7.0 ng g^−1^ and 15.5 ng g^−1^ (He et al. 2022). Laboratory tests have shown that pendimethalin at field concentrations has no effect on soil invertebrates (Belden et al. 2005), including larvae of the green lacewing *Chrysoperla carnea* (Maia et al. 2016). However, nine times higher levels of the recommended dose for the field (1 mg kg^−1^; 4–kg active ingredient per ha) significantly affect reproduction of the springtail *Folsomia candida*, by reducing offspring, and the earthworm *E. fetida*, lowering weight and growth (Belden et al. 2005). Sublethal doses of this herbicide tested under laboratory conditions interfere with moulting, hatching and sexual maturity of the springtails *Cyphoderus javanus* (Chakravorty et al. 2015) and *Xenylla whelchi* (Haque et al. 2011). In addition, concentrations of oryzalin (0.24 kg a.i. per ha) and pendimethalin (1.68 kg a.i. per ha) recommended for field use and applied under laboratory conditions are considered moderately toxic to the ectoparasitoid wasp *Tiphia vernalis*, causing male mortality (Oliver et al. 2009).

In the carabid *Pterostichus melas italicus*, previous studies demonstrated that a field dose of pendimethalin (1320 gr ha^−1^) alters the profile of haemolymph peptides involved in the immune responses and in the homeostasis control, regulating other biological treats such as release of digestive enzymes in the midgut, myostimulation of the dorsal vessel, gut and reproductive system (Aiello et al. 2022), as well as the climax of microbial communities in the gut (Giglio et al. 2021). Because the gut microbiota is involved in a wide range of physiological and metabolic activities (nutrition, detoxification, resistance to pathogens), these results suggest that exposure to this class of herbicides is likely to affect interspecific relationships, with serious implications for the fitness and survival of organisms and ecological role of species.

In vertebrates, chronic exposure to dinitroaniline leads to multigenerational transmitted effects (Fig. 1). Continuous exposure of zebrafish to 2-bromo-4,6-dinitroanilin causes alterations in gene transcription in the early life stages and reproductive dysfunction in the F1 generation that result in adverse developmental effects in the F2 generation (Xie et al. 2019). Adults of rainbow trout (*Oncorhynchus mykiss*) exposed to 0.85 μg L^−1^ pendimethalin for 18 months showed cumulative transgenerational and direct effects on both larval development and offspring pathogen susceptibility (Danion et al. 2018).

Current data on epidemiological studies evaluating human health effects are limited and sparse, with two studies on lung cancer risk associated with exposure to pendimethalin in farmers and nursery workers (Alavanja et al. 2004; Hou et al. 2006).

## Conclusive remarks on the multilevel effects

The intensification of herbicide use in recent decades has led to an increase in data demonstrating its adverse effects on non-target species including humans and ecosystems. The information reported in this review highlights that although dinitroanilines act primarily in plants, they can affect the cellular compartments of animals causing various physiological, metabolic, morphological and behavioural alterations in organisms. Toxicity depends on the concentration of the active ingredient or commercial formulation tested on different cell types or model species in vitro and in vivo studies, respectively. The variability of short-term toxic effects recorded in in vivo tests on both invertebrates and vertebrates likely depends on the species-specific ability of organisms to uptake contaminants through oral and contact exposure and to excrete them by detoxification mechanisms.

**Fig. 2:**
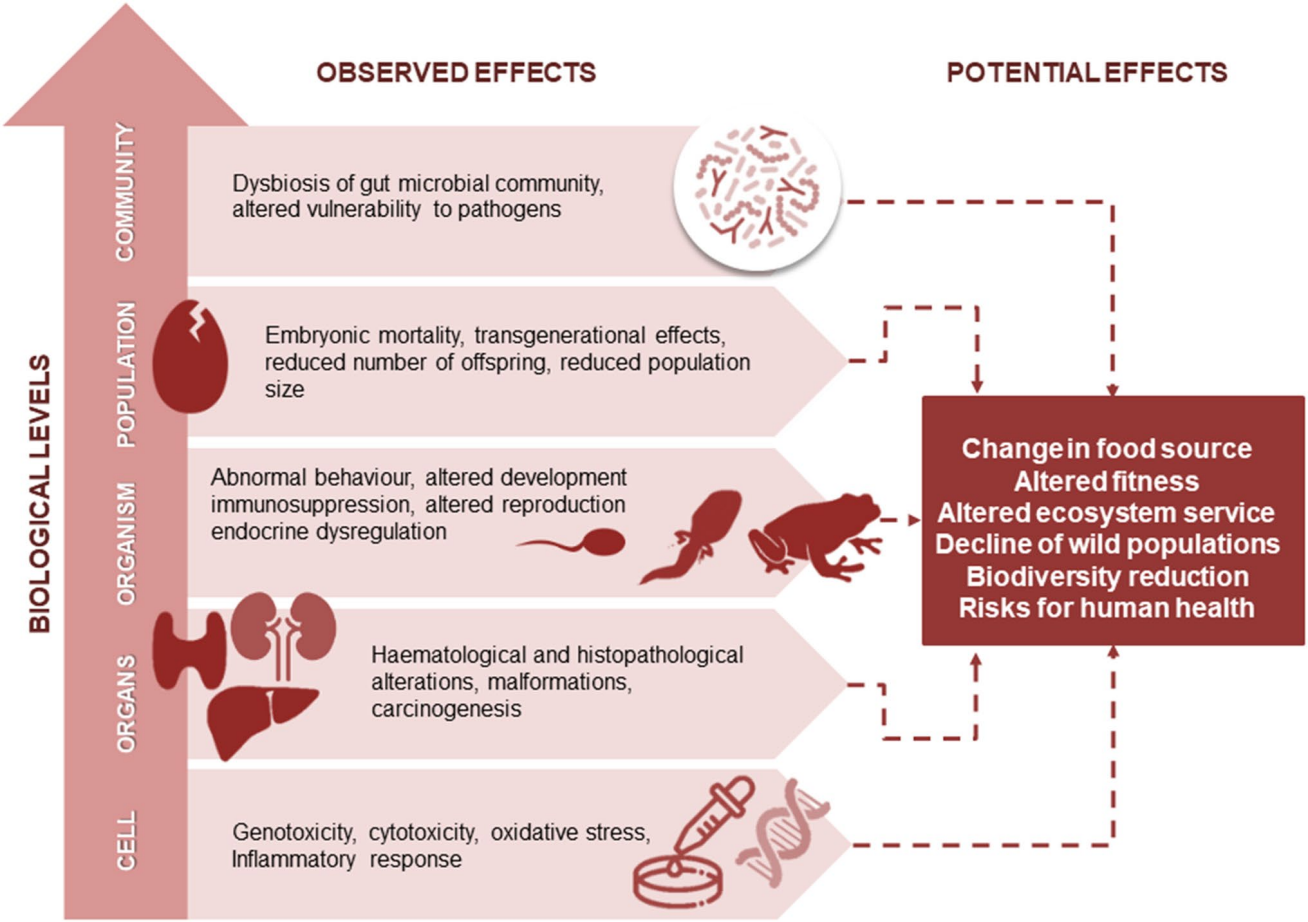
Schematic diagram of observed and potential side effects of dinitroanilines at multiple biological levels

Sublethal effects are found at all levels of biological organization (Fig. 2), even if the mechanism of action on the animal cellular compartment is unclear and can only be supposed. Resistance to dinitroaniline, found in protozoa, plants, and algae due to mutations in the tubulin primary structure (Thr239 in *Eleusine indaca* and Tyr24 or Lys350 in *Chlamydomonas reinhardtii*) (Lee and Huang 1990; Anthony and Hussey 1999), may suggest the potential non-specific binding of these herbicides to conserved sites (e.g. Lys350 in beta tubulin of higher eukaryotes including animal cells) resulting in slight changes in the primary structure of proteins that impair their biological activity in non-target organisms. This could be one of the main factors for sub-lethal effects such as cytotoxicity and genotoxicity that occur at the cellular level in both in vivo and in vitro exposure tests for different species and cell lines. Dinitroanilines induce an inflammatory response involving the oxidative stress pathway (Park et al. 2021) and alterations in the nuclear compartment, such as micronuclei, chromosomal aberrations (Ribas et al. 1996; Kılıç et al. 2018), DNA damage and an increase in apoptosis (Ansari et al. 2018; Park et al. 2021), which are probably related to the ability of these molecules to bind DNA (Ahmad et al. 2016). Changes in tissue structures and enzyme activities (Tabassum et al. 2016; Ansari et al. 2018) in the liver, excretory system, gills and thyroid suggest that these organs are the main targets of dinitroanilines in vertebrates (Leonard et al. 2019; Strupp et al. 2020b), likely due to their affinity for lipid-rich tissues. The ability of dinitroanilines to form a complex that binds tubulin in the cytoplasmic compartment and to disrupt the membrane ion transport mechanism may contribute to alter cellular homeostasis by triggering functional and morphological changes at the organism level. The resulting effects involve different life-traits such as growth, development, longevity, resistance to pathogens and reproduction. Effects on embryonic and postembryonic development in terms of growth (Travlos et al. 2017), moulting, metamorphosis, sexual maturity (Chakravorty et al. 2015) and malformations and aberrant or incomplete organogenesis (Strupp et al. 2020b; Wang et al. 2022) have been noted in several species, mainly from aquatic habitats. Dinitroanilines interfere with the endocrine network, by interacting with oestrogen and androgen receptors (Gupta and Verma 2020), potentially affecting gametogenesis, reproduction and fitness of individuals as well as interfering with the thyroglobulin synthesis (Pan et al. 2004), which is directly responsible for thyroid function (Teixeira et al. 2020), and indirectly involved in various functions, such as metabolism (Pucci et al. 2000), growth (Berdanier 2003) and fecundity (Karaca and Akpak 2015). In light of these findings, the observed reduction in the number of offspring (Belden et al. 2005) and transgenerational effects in the form of developmental and fertility alterations (Danion et al. 2018) should be considered as a potential warning tool for the risks associated with continuous exposure and long-term effects of these compounds.

The relationship between sublethal effects on cells and organisms and the implications on populations and communities (Fig. 2) has not been well studied. Nevertheless, this class of herbicides interacts with the immune system leading to changes in haematological parameters, such as leukopenia in vertebrates (Danion et al. 2012), and reductions in circulating cells and antimicrobial activity in insects (Giglio et al. 2019; Vommaro et al. 2021). The consequence of immunosuppression could be a reduced ability of the host to cope with pathogens, leading to lower survival and fitness of individuals and, consequently, to negative population effects. Dinitroanilines appear to interfere with important interspecific relationships within the community, such as host–pathogen interaction (Danion et al. 2018) and mutualistic association with the gut microbiota (Giglio et al. 2021), impairing physiological functions such as metabolism, immune response and development, as well as community structure and composition.

## Perspectives for future research

The studies conducted so far seem to indicate a systemic effect of dinitroaniline exposure, which impacts multiple biological levels and diverse organs and functions, with potential and underestimated consequences for wild species, alters population and community dynamics and poses a risk to biodiversity and human health. This raises the question of whether exposure to these herbicides, which accumulate in soil over time as a result of continued applications, can be harmful and have potential direct (feeding on contaminated prey or ingestion through contact) and indirect (depletion of food) effects on population of non-target species, which provide ecosystem services by recycling nutrients, controlling pests and regulating interspecific relationships (prey-predator, host–pathogen, mutualistic association) in the environmental system. In addition, because some metabolic pathways, such as oxidative phosphorylation, and cellular mechanism, such as phagocytosis, are ubiquitous additional analyses are needed to clarify the effects of chronic exposure to humans through direct contact or daily ingestion of residual doses in food (He et al. 2020). The cytotoxic effects observed in pharmacokinetic tests should be carefully considered to prevent toxic effects from the application of the newest chemotherapies using some dinitroaniline (Casino et al. 2018; de Souza et al. 2018) as antiparasitic drugs against protozoa that cause various human diseases such as malaria, toxoplasmosis and leishmaniasis. Most acute toxicity tests have been carried out using the technical grade of an active ingredient. Thus, there is a clear need for further studies to evaluate the effects of commercial formulations that are enriched with co-formulates to make the active ingredient more stable and active over a longer period of time (Mesnage and Antoniou 2018), analogously to what has been demonstrated for other classes of compounds (Costas-Ferreira et al. 2022; Da Silva et al. 2022). Based on the principle of three Rs—Replacement, Reduction and Refinement—we suggest the use of the yeast *Saccharomyces cerevisiae* as an alternative eukaryotic model for future toxicological studies (Braconi et al. 2016). Furthermore, the persistence of these substances in soil requires that exposure time and sublethal effects of residual doses must be considered for a comprehensive ecological risk assessment. The studies presented here reinforce the warning that herbicides, although important to agriculture, should be used with greater caution, applied at recommended rates and replaced, as much as possible, with other environmentally and biodiversity-friendly methods.

## Data Availability

Not applicable.
